# Isolation and Heterologous Expression of a Polygalacturonase Produced by *Fusarium oxysporum* f. sp. *cubense* Race 1 and 4

**DOI:** 10.3390/ijms16047595

**Published:** 2015-04-03

**Authors:** Zhangyong Dong, Zhenzhong Wang

**Affiliations:** 1 Department of Plant Protection, Zhongkai University of Agriculture and Engineering, Guangzhou 510225, China; E-Mail: dongzhangyong@zhku.edu.cn; 2 Laboratory of Physiological Plant Pathology, South China Agricultural University, Guangzhou 510642, China

**Keywords:** polygalacturonase, *Fusarium oxysporum* f. sp. *cubense*, Fusarium wilt, banana

## Abstract

Fusarium wilt (Panama disease) caused by *Fusarium oxysporum* f. sp. *cubense* (FOC) represents a significant threat to banana (*Musa* spp.) production. *Musa* AAB is susceptible to Race 1 (FOC1) and Race 4 (FOC4), while Cavendish *Musa* AAA is found to be resistant to FOC1 but still susceptible to Race 4. A polygalacturonase (PGC3) was purified from the supernatant of *Fusarium oxysporum* f. sp. *cubense* race 4 (FOC4), which is the pathogen of Fusarium wilt. PGC3 had an apparent molecular weight of 45 kDa according to SDS-PAGE. The enzyme hydrolyzed polygalacturonic acid in an exo-manner, as demonstrated by analysis of degradation products. The *K*_m_ and *V*_max_ values of PGC3 from FOC4 were determined to be 0.70 mg·mL^−1^ and 101.01 Units·mg·protein^−1^·min^−1^, respectively. Two *pgc3* genes encoding PGC3 from FOC4 and FOC1, both genes of 1368 bp in length encode 456 amino-acid residues with a predicted signal peptide sequence of 21 amino acids. There are 16 nucleotide sites difference between FOC4-*pgc3* and FOC1-*pgc3*, only leading to four amino acid residues difference. In order to obtain adequate amounts of protein required for functional studies, two genes were cloned into the expression vector pPICZaA and then expressed in *Pichia pastoris* strains of SMD1168. The recombinant PGC3, r-FOC1-PGC3 and r-FOC4-PGC3, were expressed and purified as active proteins. The optimal PGC3 activity was observed at 50 °C and pH 4.5. Both recombinant PGC3 retained >40% activity at pH 3–7 and >50% activity in 10–50 °C. Both recombinant PGC3 proteins could induce a response but with different levels of tissue maceration and necrosis in banana plants. In sum, our results indicate that PGC3 is an exo-PG and can be produced with full function in *P. pastoris.*

## 1. Introduction

Banana (*Musa* spp.) is the world’s principal fruit and is regarded as the fourth most important crop in developing countries [1]. It suffers from several devastating diseases, and the most famous one is Fusarium wilt or Panama disease, which caused by the fungus *Fusarium oxysporum* f. sp. *cubense* (FOC) and is widely regarded as one of the most destructive plant diseases in the world [2,3].

Earlier last century, a physiological race name FOC1 previously infected the cultivar Gros Michel, which was the main exported banana variety in the 1950s. Consequently, the Gros Michel was replaced by the Cavendish variety, which is resistant to FOC1. But Cavendish is susceptible to the new FOC4 strain, which is capable of attacking almost all the banana varieties [4]. Until recently, FOC4 had been recorded to cause serious losses in the regions of Asia, Australia and Africa. Grimm (2008) worried that if FOC4 hit the banana heartland in Latin America, it would be devastating for banana [5].

The plant cell wall is a formidable barrier to plant pathogens. Plant pathogenic fungi produce an array of cell wall-degrading enzymes (CWDEs) that play significant roles throughout the fungal life, including acquisition of nutrients and decomposition of plant cell walls, and may be important in pathogenicity [6]. Among them, polygalacturonases (PGs) are often the first enzymes secreted by pathogens growing on the plant cell walls [7] and may play a key role since their action on pectin makes other cell wall components more accessible to other CWDEs [8].

Exopolygalacturonases (exo-PGs) have been studied in the fungal plant pathogen *Cochliobolus carbonum* [9] and *Fusarium oxysporum* f. sp. *lycopersici* [10] concerning their role in disease. Endo-PGs (EC 3.2.1.15) cleave the backbone of polygalacturonan internally, whereas exo-PGs (EC 3.2.1.67) hydrolyze monomers progressively from the non-reducing end of the substrate. Exo-PGs may have an important function in pathogen-plant interactions, since they degrade elicitor-active oligogalacturonides released by endo-PGs [11] and are generally not inhibited by plant polygalacturonase-inhibiting proteins (PGIPs) [12]. Schacht, T. *et al.*, (2011) found that the inhibition of endo-PG was higher than the inhibition of exo-PGs by PGIPs extraction from Ralstonia solanacearum-inoculated plants, and noted that through higher expression of exo-PGs the pathogen might effectively escape plant defense responses through late or no recognition by the plant, since potent elicitor-active oligomers were not produced or could be rapidly degraded by the action of exo-PGs [13]. Although some previous studies have been conducted, understanding of exo-PGs is limited. In the previous studies, an endo-PG (PGC1) and an exo-PG (PGC2) have been characterized and cloned in FOC4 (GenBank Accession no. GI:223960661 and GI:281372497).

In this study, we report the isolation and the purification of an exo-PG (PGC3) from the pathogen FOC4. We cloned the *pgc3* genes of FOC1 and FOC4 and then expressed in *P. pastoris*. Both of them had exo-PG activity. Further studies are required to obtain insight into the role of PGC3 in FOC pathogenicity in banana cultivars.

## 2. Results and Discussion

### 2.1. Purification of an Exo-Polygalacturonase (PGC3) from Fusarium oxysporum f. sp. cubense Race 4 (FOC4)

The PG specific activity in the FOC4 culture supernatant could be found when the fungus was grown in culture in the presence of citrus pectin. PGC3 was purified from FOC4 through successive steps of ultra-filtration, gel filtration chromatography and cation exchange chromatography. After the purification process, the total protein was 0.4 mg and the total activity was 7.25 Units. The specific activity increased from 3.586 to 18.125 Units·mg·protein^−1^·min^−1^ (Table 1). A faint single peak of PG activity was observed when culture of FOC4 was applied to a Sephacryl S-100 gel filtration column. Then a significant single peak of PG activity can be seen after subjecting the pooled PG fraction to a cation exchange chromatography (Sepharose FF CM). We found some proteins during a SDS-PAGE analysis on the whole fractions of this single peak of PG activity (tube No. 9 to 23). When we tried to concentrate them in a single collection tube and then run the SDS-PAGE analysis, we found a single band and high PG activity of the tube No. 12.

**Table 1:** Purification of PGC3 from culture of FOC4 grown on potato dextrose broth (PDB) supplemented with 1% citrus pectin.

Step	Total Protein (mg)	Total Activity (U ^a^)	Yield (% activity)	Specific Activity (U ^a^/mg)
Crude	36.6	131.2476	100	3.586
Ultrafiltration	8.21	70.2604	53.53	8.5579
Sephacryl S-100	1.85	26.35	20.08	14.2432
Sepharose FF CM	0.4	7.25	5.52	18.125

^a^ One U of PG activity was defined as the amount of enzyme that liberated one µmole of GA in one minute at 50 °C.

**Figure 1:**
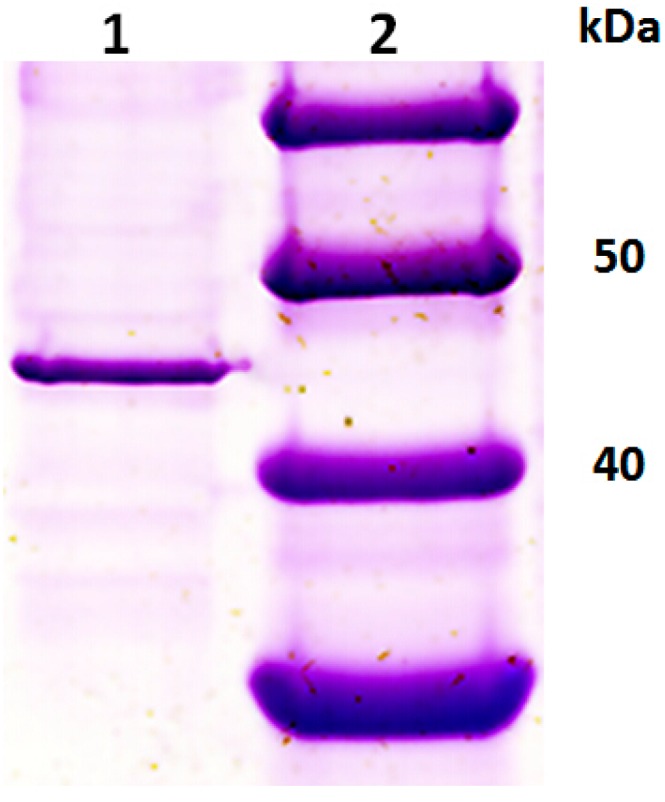
SDS-PAGE analysis of purified PGC3 from FOC4. Lane 1: purified PGC3; Lane 2: protein marker.

The sample application on SDS-PAGE showing one single protein band indicated that PGC3 was purified to homogeneity and had a molecular weight of 45 kDa according to the markers (Figure 1), which was different from PGC2 (63 kDa) purified previously [14]. It is obvious that there is more than one PG in FOC4 like other pathogenic fungus. Two PGs in *Fusarium graminearum* were characterized during infection of wheat spikelets [15] and three PGs in *Fusarium oxysporum* f. sp. *lycopersici* were purified and characterized and two of them were exo-PGs [16,17,18].

The purified PGC3 was sent for *N*-terminal sequencing and 15 residues sequence was obtained as TSSRNSALPKRPHVE. BlastP program was run with *N*-terminal sequence of PGC3 to the National Center for Biotechnology Information (NCBI) non-redundant protein sequences (Nr) database, the top match (100%) was exopolygalacturonase from *Fusarium oxysporum* f. sp. *fragariae* (BAE97149.1).

### 2.2. Isolation of Genes Encoding PGC3 from FOC1 and FOC4

The full-length cDNA of *pgc3* from FOC4 was cloned by RACE PCR according to the *N*-terminal sequence of PGC3. The specific primers were then designed to amplify the full-length cDNA of *pgc3* from FOC1 and the DNA sequences of *pgc3* from both FOC1 and FOC4. A full-length 1622 nucleotide DNA sequence was isolated and sequenced from FOC1 and FOC4. Sequencing revealed the presence of an open reading frame (ORF) of 1368 nucleotides, interrupted by five introns of 59, 47, 47, 51 and 50 nucleotides, and encoding a predicted protein of 456 amino. Analysis with SignalP detected a putative *N*-terminal signal peptide sequence of 21 amino acids that when cleaved would produce a mature protein. The deduced mature protein of *pgc3* from FOC1 (FOC1-*pgc3*) and *pgc3* from FOC4 (FOC4-*pgc3*) had the calculated molecular mass of 47.88 and 47.95 kDa, the calculated isoelectric point (pI) of 5.97 and 5.79, respectively. PG (*pgx4*) with very similar calculated molecular mass of 47.9 kDa but different pI (8.0) studying the fungus FOL 4287. We are not sure if a predicted program leads to the different pI or some unknown reasons [10].

**Figure 2:**
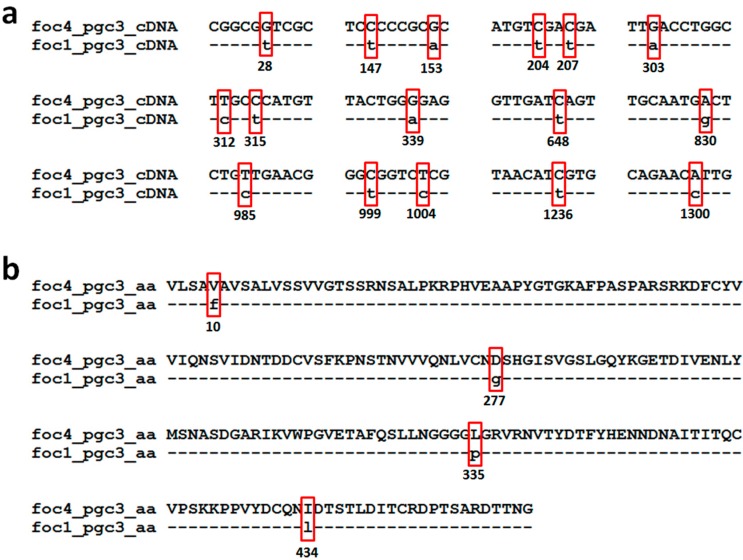
Pairwise sequence alignment of the nucleotide sequences (open reading frame, ORF) and predicted proteins of *pgc3* from FOC1 and FOC4. (**a**) Alignment of ORF of *pgc3* from FOC1 and FOC4; (**b**) alignment of predicted proteins of *pgc3* from FOC1 and FOC4.

The FOC4-*pgc3* shared as low as 41.5% nucleotide sequences identity with FOC4-*pgc2*, while their amino acid identity was only 28.9%. There are 16 nucleotide sites difference between FOC4-*pgc3* and FOC1-*pgc3* as following 28, 147, 153, 204, 207, 303, 312, 315, 339, 648, 830, 985, 999, 1004, 1236, and 1300 (Figure 2a). The FOC1-*pgc3* shared as high as 99.12% amino acid sequence identity with FOC4-*pgc3*. There are four amino acid differences between FOC4-*pgc3* and FOC1-*pgc3*, as following 10, 277, 335, and 434 (Figure 2b). Among these sites, site 277 was the putative glycosylation site, and site 335 was the *N*-myristoylation sites. We are unsure if these site variations lead to the functional difference between these two PGs.

The complete nucleotide sequences of *pgc3* genes from FOC1 and FOC4 were deposited in the GenBank database under accession numbers KP768396 and KP768397.

### 2.3. Expression and Purification of Recombinant PGC3

**Figure 3:**
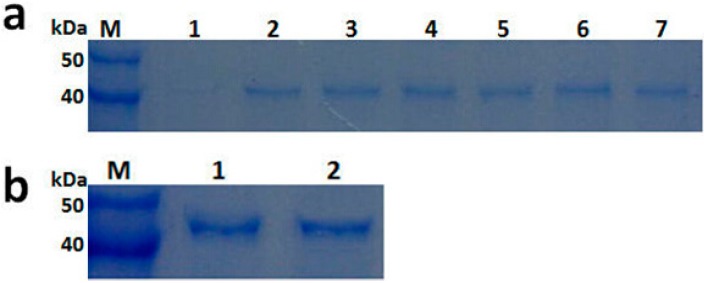
SDS-PAGE analysis of recombinant PGC3 from FOC1 and FOC4 produced in *P. pastoris*. (**a**) Cultures were induced with methanol as described in Experiment Section and supernatants from 1, 2 to 3 day were collected. Lane M: protein marker; Lane 1: cultures transformed with pPICZaA; Lane 2–4: culture supernatant from FOC1 at 1, 2, and 3 day; Lane 5–7: culture supernatant from FOC4 at 1, 2, and 3 day; (**b**) SDS-PAGE analysis of purified PGC3 from FOC1 and FOC4 produced in *P. pastoris*. Lane M: protein marker; Lane 1: purified r-FOC1-PGC3; Lane 2: purified r-FOC4-PGC3.

Recombinant PGC3 from FOC1 and FOC4 were successfully produced as secreted proteins r-FOC1-PGC3 and r-FOC4-PGC3 using expression vector pPICZαA and the yeast *P. pastoris* SMD1168. Culture samples taken at 1, 2 and 3 days post-induction were analyzed by SDS-PAGE (Figure 3). Proteins of about 45 kDa were detected from the r-FOC1-PGC3 and r-FOC4-PGC3 transformant cultures, but were not observed in control *P. pastoris* transformed with pPICZαA vector. After induction with methanol at two days, recombinant PGC3 were purified by concentrating with the Amicon system, accompanied by purification with the gel filtration chromatography. SDS-PAGE showed one single protein band and indicated that r-FOC1-PGC3 and r-FOC4-PGC3 were purified to homogeneity (Figure 3). The molecular weight of both recombinant proteins was about 45 kDa according to the molecular weight markers.

### 2.4. Biochemical Characterization of PGC3

Hydrolysis product and kinetic parameter analysis were performed using the purified PGC3 from the FOC4 culture. The terminal product of enzymatic hydrolysis of PGA by purified PGC3 from FOC4 was analyzed. Galacturonic acid (GA) was the only degradation product detected during enzyme activity and its hydrolysis of the 3% of substrate leading to a 16% reduction of viscosity, demonstrated that PGC3 is an exo-PG. The *K*_m_ and *V*_max_ values of PGC3 from FOC4 were determined to be 0.70 mg·mL^−1^ and 101.01 Units·mg·protein^−1^·min^−1^, respectively.

**Figure 4:**
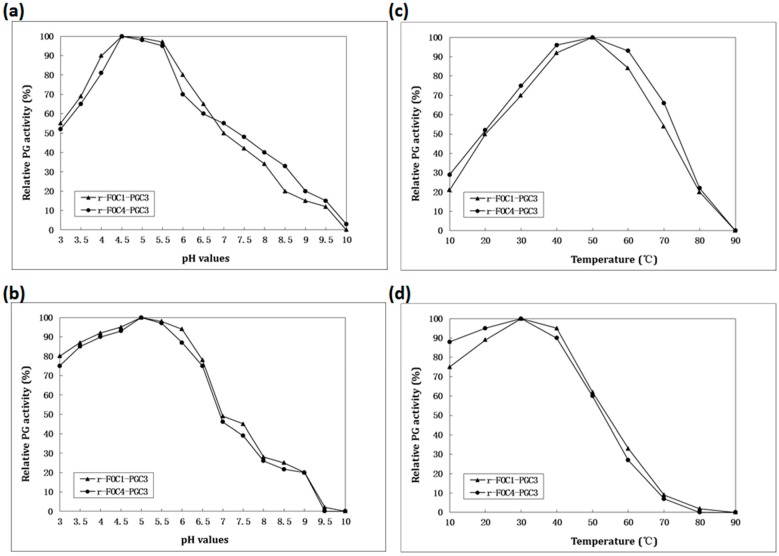
Enzymatic activity and stability. (**a**) Determination of the optimal pH; (**b**) Enzymatic stability with respect to pH; (**c**) Determination of the optimal temperature; (**d**) Enzymatic stability with respect to temperature. To determine the optimal pH, the enzyme activity was assayed using 100 mM potassium phosphate buffer for pH values between 3 and 10 at 50 °C, and 0.5% (*w*/*v*) PGA as substrates. The effect of temperature on PGC3 activity was determined in the same buffer at pH 4.5, between 10 and 90 °C. One Unit of PG activity was defined as one µmol/L of GA released by enzyme·min^−1^.To estimate the pH stability, samples were incubated in 100 mM potassium phosphate buffer of different pH values at 4 °C for 24 h. To evaluate the thermal stability, protein samples were incubated at different temperatures in the same buffer at pH 4.5, for 2 h. The residual activity was detected according to the method as previously described.

To identify the stability and optimal pH value and temperature of PGC3, recombinant PGC3 from FOC1 and FOC4 were used and compared. The optimum pH value and temperature of the recombinant PGC3 were investigated with PGA as the substrate. Both purified recombinant PGC3 from both FOC1 and FOC4 exhibited optimum activity at pH 4.5 (Figure 4a), and the highest activity at 50 °C (Figure 4b). Similarly, the optimum pH and temperature of PGC2 from FOC4 were 5 and 50 °C, respectively [14], and the pH value and temperature optima for the PG activity from PG2 of *Fusarium oxysporum* f. sp. *lycopersici* (FOL) were 5 and 55 °C [17]. The temperature optimum for the PG activity from PG3 of FOL was 55 °C and with a broad range of pH from 3.5 to 9 [18]. An exo-PG secreted by Rhizopus oryzae had an optimum temperature of 50 °C but an optimum pH of 5 which is higher than that of PGC3 [19]. The optimum temperature and pH of an exo-PG from a thermotolerant fungus *Aspergillus niveus* were 50 °C and 4–6.5, respectively [20].

To calculate the pH stability of recombinant PGC3, samples were incubated in buffers of different pH values at 4 °C for 24 h. The remaining PG activity was assayed. Both recombinant PGC3s retained more than 40% peak activity at pHs ranging from 3 to 7 (Figure 4c). To investigate the thermostability of recombinant PGC3, both recombinant PGC3 were incubated at different temperatures in 100 mM potassium phosphate buffer, pH 4.5, for 2 h. The residual activity was found. Both recombinant PGC3 retained more than 50% activity in the temperature range between 10 and 50 °C (Figure 4d). Compared to PGC2, it retained more than 70% activity at pH 3 to 6, and 50% activity at 10 to 50 °C [14]. It suggests that PGC3 has lower activity under the acid conditions than PGC2.

### 2.5. Active Recombinant PGC3 Causes Tissue Maceration and Necrosis

Purified PGC3-FOC1 and PGC3-FOC4 were inoculated with banana tissues to test their ability to macerate banana tissue. Surface sterilization of banana stem tissues were incubated with 1 Unit of recombinant PGC3 and 1 mL of 50 mM sodium acetate buffer (pH 4.5), and then macerations were evaluated after 48 h post incubation.

**Figure 5:**
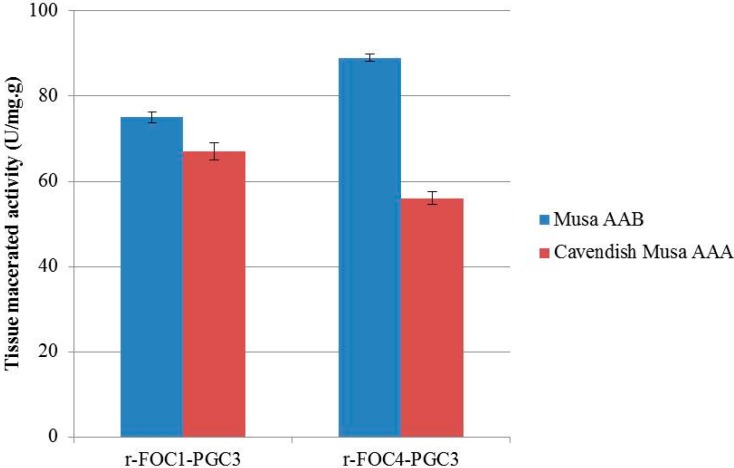
Enzyme maceration activity in banana tissue. 1 U of purified enzyme mixed with 1 mL of 50 mM sodium acetate buffer pH 4.5 was inoculated with sterilized banana tissues, and maceration was evaluated after 48 h at 50 °C. The same buffer without enzyme as negative control. The reducing sugar content was analyzed by the method of Somogyi in three replications.

The maceration activity of r-FOC1-PGC3 to *Musa* AAB was lower than that of r-FOC4-PGC3, while the maceration activity of r-FOC1-PGC3 to Cavendish *Musa* AAA was higher than that of r-FOC4-PGC3. Both these two enzymes showed higher maceration activity on *Musa* AAB than Cavendish *Musa* AAA. Similarly, both r-FOC1-PGC2 and r-FOC4-PGC2 showed higher maceration activity on *Musa* AAB than Cavendish *Musa* AAA [14]. The results suggest that pectins of *Musa* AAB and Cavendish *Musa* AAA, as an important component of cell walls, might differ in polymer structure because Cavendish *Musa* AAA pectin is a poor substrate for exo-PGs compared to that of *Musa* AAB (Figure 5).

**Figure 6:**
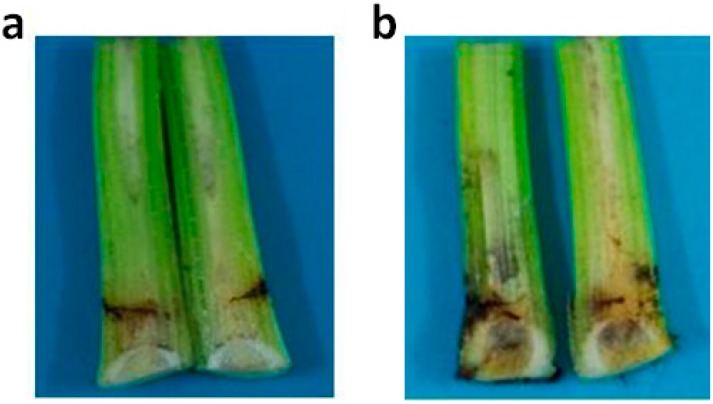
Tissue necrosis analysis of Cavendish banana stem. (**a**) Plants injected with r-FOC1-PGC3; (**b**) Plants injected with r-FOC4-PGC3. Each plant was injected with 1 U of enzyme. Each treatment was cut for observing vascular necrosis after five days.

Five days after the stems of Cavendish *Musa* AAA were injected with 1 U of recombinant PGC3, the necrosis could be observed on the stem vascular tissues. Nothing can be seen in the water control and empty vector transferred control. However, the r-FOC1-PGC3 induced more necrosis on stem vascular tissues compared to r-FOC4-PGC3 (Figure 6). As shown on the maceration activity, r-FOC1-PGC3 had higher maceration activity on Cavendish *Musa* AAA than r-FOC4-PGC3. It seems that r-FOC1-PGC3 had a higher activity than r-FOC4-PGC3. It is quite interesting that Cavendish *Musa* AAA is known to be resistant to FOC1 and susceptive to FOC4; no or less symptom should be seen after FOC1 inoculation. Nevertheless, we found purified recombinant PGC3 from FOC1 can be active towards Cavendish *Musa* AAA, and even at higher levels than that of FOC4. There might be two mechanisms for this. First, PGC3 had no or lower expression when FOC1 infected Cavendish *Musa* AAA, then less necrosis can be seen after FOC1 infection. Though both FOC1 and FOC4 were found to be able to invade banana roots and spread to root vascular tissues in the first two days following inoculation, the gene expression had not been measured for a longer time since necrosis symptoms can be seen [21]. Further, we do not know if PGC3 has gene expression when FOC1 infected Cavendish *Musa* AAA. Second, some signal factors might control FOC1 infected Cavendish *Musa* AAA. If lower amounts of FOC1 are in plants, the necrosis would be less, or nothing can be observed. However, the mechanism about this should be further confirmed.

## 3. Experimental Section

### 3.1. Fungal Strains and Growth Condition

*F. oxysporum* f. sp. *cubense* race 4 (FOC4) was obtained from Panyu, Guangzhou, China, and *F. oxysporum* f. sp. *cubense* race 1 (FOC1) was obtained from Jiangnan, Nanning, China, respectively.

For DNA extraction, mycelium was obtained from cultures grown for five days in potato dextrose broth (PDB) in flasks on a rotary shaker at 110 rpm and 25 °C. For RNA extraction, mycelium was obtained from cultures of PDB supplemented with 1% (*w*/*v*) citrus pectin. Citrus pectin was obtained from Sigma-Aldrich (St. Louis, MO, USA).

### 3.2. Enzyme Activity and Protein Measurements

Exopolygalacturonase (exo-PG) activity was assayed in a mixture (1 mL of total volume) containing 0.5% polygalacturonate (PGA) (*w*/*v*), 50 mM sodium acetate buffer (pH 4.5), and various amounts of enzyme solution at 50 °C for 30 min. The number of reducing groups, expressed as galacturonic acid (GA) released by enzymatic action, was determined by the method of previously reported protocols [22]. One unit of enzyme activity (U) was defined as the amount of enzyme that releases 1 µmol of GA per minute under the assay conditions.

The molecular weight of the purified enzyme determined by SDS-PAGE was performed using 12% acrylamide according to the method of Laemmli [23]. After electrophoresis, gels were stained with Coomassie brilliant blue G250.

Protein concentration was determined according to the method of Bradford [24], using bovine serum albumin (BSA) as the standard.

### 3.3. Enzyme Purification

Culture of FOC4 grown in PDB with 1% citrus pectin was centrifuged at 16,000× *g* for 30 min. The supernatant was collected and concentrated with an Amicon 8400 ultrafiltration system with a 30 kDa molecular weight cut-off (MWCO) membrane filter, and then applied to a gel filtration column Sephacryl S-100 from GE Healthcare Bioscience (Pittsburgh, PA, USA) equilibrated and eluted with 50 mM sodium acetate buffer (pH 4.5) at a flow rate of 1 mL/min. Fractions containing PG activity were pooled and applied to a cation exchange column (Sepharose FF CM, GE Healthcare Bioscience) equilibrated with 20 mM sodium acetate buffer (pH 4.5). The column was eluted with a gradient of NaCl (0–0.7 M) at a flow rate 2 mL/min. Each tube of PG activity peak was checked by PG activity and SDS-PAGE after concentration.

### 3.4. Enzyme Characterization

To analyze the hydrolysis products, the samples (0.02 U enzyme in 0.5 mL water) were added to 1 mL of 0.5% (*w*/*v*) PGA in 50 mM sodium acetate buffer (pH 4.5) and incubated at 50 °C, for 10, 20, 30, 40, 50, 60 min, and then used for PG activity assay. The method was referred to Somogyi’s method [23]. The method to distinguish endo- or exo-PGs is to measure the degree of viscosity and reducing sugar analysis. The 3% of the substrate being hydrolyzed by endo-PGs can lead to 50% reduction of viscosity, while exo-PGs need 20% of the substrate being hydrolyzed.

The Michaelis constant (*K*_m_) and *V*_max_ values were determined from Lineweaver–Burk plots of enzyme activity measured with the PGA as substrates, at concentrations between 0.25% and 1.25% at optimum pH and temperature, and then plotted the results.

To determine the optimal pH, the PG activity was assayed using 100 mM potassium phosphate buffer for pH values between 3 and 10 at 50 °C, and 0.5% (*w*/*v*) PGA as substrates. The consequence of temperature on PG activity was determined in 100 mM potassium phosphate buffer at pH 4.5, between 10 and 90 °C. To calculate the pH stability, the samples were covered in 100 mM potassium phosphate buffer of different pH values at 4 °C for 24 h, and then used for PG activity assay. To assess the thermal stability, the samples were covered in 100 mM potassium phosphate buffer at pH 4.5 for 2 h, between 10 and 90 °C, and then used for PG activity assay. Three replicates were tested per treatment as well as the negative control.

### 3.5. Cloning of Fungal pgc3 Genes

For Race PCR, cDNA of FOC4 was synthesised by the RNA with murine leukemia-virus reverse transcriptase (TaKaRa, Tokyo, Japan). Two primers were designed based on the *N*-terminal amino acid sequence of PGC3. 3'-RACE PCR were conducted using the primer *pgc3*-P1: 5'-NWSNWSNMGNAAYWSNGC-3', and *pgc3*-P2: 5'-TNCCNAARMGNCCNCAYG-3', together with Oligo(dT)20. Amplification conditions were one cycle of 3 min at 94 °C, 35 cycles of 94 °C for 30 s, 52 °C for 30 s, and 72 °C for 90 s, then 10 min for 72 °C. The amplified fragments were purified and sequenced.

Based on the sequence of RACE PCR, the gene sequences and coding sequences of FOC1 and FOC4 were obtained by RT-PCR using the primers (5'-GAATTCATGTTGTTCAATAACGTTCTTT-3') and (5'-TCTAGATCCATTGGTTGTGTCCCTG-3'). The *Eco*RI and *Xba*I restriction sites are underlined. Amplification conditions were one cycle of 3 min at 94 °C, 35 cycles of 94 °C for 30 s, 55 °C for 30 s and 72 °C for 90 s, then 10 min for 72 °C. The RT-PCR products were purified and then digested with *Eco*RI and *Xba*I and subcloned into the same enzymes digested pPICZaA (Life Technologies, Grand Island, NY, USA) to generate recombinant eukaryotic expression vector, pPICZaA-*pgc3*-FOC1 and pPICZaA-*pgc3*-FOC4. The presence of the inserted *pgc3*-FOC1 and *pgc3*-FOC4 genes was verified by sequencing.

The complete gene sequences of *pgc3* from FOC1 and FOC4 were amplified and sequenced using DNA of FOC1 and FOC4 as the template and primers as mentioned above.

### 3.6. Expression and Purification of Recombinant Enzymes in P. pastoris

Yeast transformation was performed according to the manufacturer’s instructions. The recombinant plasmid pPICZaA-*pgc3*-FOC1 and pPICZaA-*pgc3*-FOC4 were linearized by digestion with *Sac*I and transformed into *P. pastoris* SMD1168 strain by electroporation. The SMD1168 strains transformed with or without pPICZaA plasmid served as negative controls. The cells were incubated in yeast extract peptone dextrose (YPD) plate containing 1% yeast extract, 2% peptone, 2% dextrose, and 100 µg/mL of Zeocin at 28 °C for 48 h. The integration of the *pgc3* gene into SMD1168 was determined by PCR using 5'AOX1 and 3'AOX1 primers, which can be found in the instruction of pPICZaA.

Yeast transformants were grown in 30 mL of BMGY medium (1% yeast extract, 2% peptone, 1.34% yeast nitrogen base, 100 mM potassium phosphate, 4 × 10^−5^% biotin and 1% glycerol) at 28 °C for 24 h, and then transferred to 200 mL BMMY medium (1% yeast extract, 2% peptone, 1.34% yeast nitrogen base, 100 mM potassium phosphate, 4 × 10^−5^% biotin and 0.5% methanol) at 28°. The samples were collected at 1, 2 and 3 day, followed by 1 mL of 100% methanol added daily to maintain the final concentration of methanol at 0.5%.

To purify the recombinant PGC3, two days induced culture was collected and centrifuged at 10,000× *g* for 20 min at 4 °C. The supernatant was concentrated by the Amicon system with a 30 kDa MWCO membrane filter, and then applied to a gel filtration column (Sephacryl S-100, Pharmacia) equilibrated and eluted with 50 mM sodium acetate buffer (pH 4.5) at a flow rate of 1 mL/min. Fractions containing PG activity were collected.

### 3.7. Tissue Maceration and Necrosis Assayed with Recombinant Enzymes

To evaluate tissue maceration, Cavendish *Musa* AAA (resistant to FOC1 and susceptible to FOC4), and *Musa* AAB (susceptible to FOC1 and FOC4) were used. A length of 1 cm tissue (~0.5 g) was taken from the healthy stems of the four leaf stage plants and placed into test tubes. The sterilized plant tissue was treated with single U of purified enzyme in 1 mL of 50 mM sodium acetate buffer (pH 4.5), and then evaluated macerated activity at 48 h post treatment. A negative control contained the same buffer instead of the enzyme. Tissue macerated activity was calculated from standards of PGA after incubation 48 h at 50 °C by the method of Somogyi [22].

For the tissue necrosis assay, one U of enzyme was injected directly to the stems of healthy banana plant. Each treatment had ten replicates. Autoclaved water and 50 mM sodium acetate buffer (pH 4.5) were used as negative controls Plant stems were cut (vertical-sectioned) to observe vascular necrosis at 5 days post treatment.
